# Acoustic build-up in on-chip stimulated Brillouin scattering

**DOI:** 10.1038/srep13656

**Published:** 2015-09-04

**Authors:** C. Wolff, M. J. Steel, B. J. Eggleton, C. G. Poulton

**Affiliations:** 1 Centre for Ultrahigh bandwidth Devices for Optical Systems (CUDOS); 2 School of Mathematical and Physical Sciences, University of Technology Sydney, NSW 2007, Australia; 3 MQ Photonics Research Centre, Department of Physics and Astronomy, Macquarie University, NSW 2109, Australia; 4 Institute of Photonics and Optical Science (IPOS), School of Physics, University of Sydney, NSW 2006, Australia

## Abstract

We investigate the role of the spatial evolution of the acoustic field in stimulated Brillouin scattering processes in short high-gain structures. When the gain is strong enough that the gain length becomes comparable to the acoustic wave decay length of order 100 microns, standard approximations treating the acoustic field as a local response no longer apply. Treating the acoustic evolution more accurately, we find that the backward SBS gain of sub-millimetre long waveguides is significantly reduced from the value obtained by the conventional treatment because the acoustic mode requires several decay lengths to build up to its nominal value. In addition, the corresponding resonance line is broadened with the development of side bands. In contrast, we argue that intra-mode forward SBS is not expected to show these effects. Our results have implications for several recent proposals and experiments on high-gain stimulated Brillouin scattering in short semiconductor waveguides.

In recent years there has been a great deal of research interest in Stimulated Brillouin Scattering (SBS), in which photons in a waveguide interact coherently with mechanical vibrations along the waveguide’s length^1^, which are induced by the light itself. Although traditionally viewed as an effect seen in long lengths (say tens of metres to kilometres) of optical fibre^2^, interest in SBS has more recently turned to on-chip implementations^3^. On-chip SBS allows photonic integration of a broad range of interesting and potentially important applications, including high-speed analog signal processing^4^,^5^,^6^, optical pulse generation^7^ and optical pulse delay^8^ as well as providing an alternate route to nano-optomechanics^9^,^10^,^11^. However, harnessing SBS in the short length scales of an optical chip is extremely challenging, because the narrow linewidth of SBS (typically <100 MHz) precludes the use of the ultra-short, high peak-power pulses prevalent in other areas of nonlinear optics. Therefore waveguides of several centimetres, relatively long by the standards of other effects in integrated nonlinear optics, are needed to establish a measurable amount of gain. Nevertheless, on-chip SBS has been demonstrated in several platforms, including chalcogenide glass waveguides^10^, as well as in silicon/silicon nitride suspended structures^12^, silicon pedestal waveguides^13^ and high-Q resonators^14^,^15^.

Miniaturization and (desirably CMOS-compatible) on-chip integration is one of the main themes in the current research on SBS. A recent hallmark of this effort was the realization that radiation pressure can significantly enhance SBS in sub-micron high index contrast waveguides, specifically silicon nanowires suspended in air^16^. There are several potential approaches to building such nanowires, including suspended membranes^12^ and pedestal waveguides Ref. 13. It is important to consider whether all approaches are equally promising, or whether some may face peculiar challenges. In particular, suspended pedestal waveguides can extend for centimeters, but to avoid sagging, membrane structures are limited to lengths of a fraction of a millimetre. So for fabrication and handling reasons, waveguides of the latter class would likely be composed of concatenations of very short units (a few hundred microns), each possessing a relatively small individual gain. If one assumes that the SBS gain of the cascaded device is simply the sum of the gains of the individual gain elements, then in principle a significant overall gain should be able to be achieved. However in current experiments on cascaded structures the measured gain remains well below expected values^16^, and it is therefore important to rigorously re-examine the expected of the SBS gain in these short waveguides.

In this paper, we show that the propagation of sound can drastically reduce the SBS-gain of any very short waveguide. High frequency acoustic waves in solids are strongly damped and so there are two length scales in SBS interactions: the acoustic decay length 1/*α*, which is of the order of tens of microns in all relevant waveguide structures from glass fibres to silicon waveguides, and the characteristic gain length 1/*G*. In traditional fibre systems, the latter is at least 10 s to 100 s of metres and the two scale lengths are separated by at least four orders of magnitude. The acoustic field can thus be considered to adiabatically follow the evolution of the optical fields. However, in the recent high gain integrated waveguides, we approach the regime where the two scale lengths are of comparable size. In this case, the “slaving” of the acoustic field to the optical fields is no longer appropriate and a more accurate accounting of the acoustic dynamics is required. This is our objective here.

Silicon nanowires motivate our work because they are the first structures in the literature where the detailed acoustic evolution might play a role, but our work applies to any short waveguide. We start with general coupled-mode theory and make no assumptions about material properties. In backward SBS and a special type of forward SBS (more specifically: inter-mode forward SBS), the acoustic group velocity is large and the sound wave needs of the order of 100*μ*m to build up as illustrated in Fig. 1. This is an important fact, because this length scale of 100*μ*m is also the maximal length over which a nanowire can be suspended in a standard SOI process without having too much to worry about sagging and it touching the silicon substrate. The acoustic build-up effectively shortens the waveguide for SBS-purposes and results in a reduction of the total SBS response over the waveguide. We show how the acoustic decay length can be estimated from the acoustic wave vector and the mechanical quality factor. Further symptoms of this effect are a broadening of the SBS-resonance and the appearance of side bands. In conventional (intra-mode) forward SBS, the acoustic group velocity vanishes and no such effect is expected. This difference of the acoustic build-up in very short waveguides may contribute to the explanation of why to date no backward SBS has been observed in suspended silicon systems that exhibit significant forward SBS^12^. We note that other factors such as nonlinear losses would also be expected to play a role in the effectiveness of SBS processes. We have recently investigated this point^17^ and found that nonlinear losses are indeed important in certain regimes. A key conclusion however is that forward and backward SBS are impacted in identical fashions for moderate Stokes power, and so cannot account for the particular challenge of observing backward SBS in silicon waveguides. For this reason, we neglect these effects in the current work allowing us to obtain analytic solutions which highlight the particular physics role of acoustic build-up. A complete numerical model would ultimately be required to account for all these effects.

## Approach

We focus on the case that the SBS-waveguide is operated as a linear small-signal amplifier for a quasi-stationary Stokes wave. This implies an undepleted pump approximation and is justified because an individual amplifier can be expected to provide at most of the order of 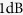 of gain in the cascaded arrangement outlined above. The following discussion can be derived from any formulation of waveguide coupled-mode theory. For consistency among our publications and for convenience, we choose the notation introduced in our recent theory paper^18^; *a*^(1)^(*z*), *a*^(2)^(*z*) and *b*(*z*) are the envelope functions of the optical Stokes wave, the input pump and the acoustic wave, respectively. The optical and acoustic frequencies are denoted by *ω* and Ω, the individual modes’ normalization powers with the symbol 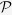. The symbol *C* denotes the inter-mode coupling parameter, which for optically lossless materials is computed from the optical eigenmodes (superscripts 1 and 2 refer to Stokes and pump modes in analogy to the optical envelopes) described via the modal electric fields *e*^(1/2)^ and modal electric induction fields *d*^(1/2)^, and the acoustic eigenmode described by the mechanical displacement field *u* via the expression
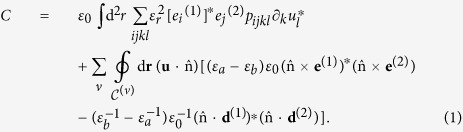


The first integral, which extends over the whole waveguide cross-section, describes the photoelastic coupling through the photoelastic (Pockels) tensor distribution *p*_*ijkl*_(**r**), where *ε*_*r*_(**r**) is the relative dielectric function and *ε*_0_ is the absolute permittivity of vacuum. The rest is the sum of contour integrals over each material discontinuity, enumerated by the index *ν*, where 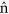 denotes the unit normal vector of the *ν*-th contour 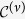 and points from the material with relative permittivity *ε*_*a*_ into the material with relative permittivity *ε*_*b*_. The optical and acoustic eigenmodes are normalized to (in principle arbitrary) power units
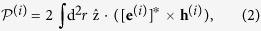

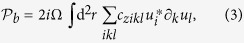
where both integrals cover the entire transverse plane and *c*_*ijkl*_ (**r**) denotes the mechanical stiffness tensor distribution.

The acoustic damping coefficient *α* is the inverse decay length, i.e. the amplitude of a sound wave decays to exp(−1) over the length of *α*^−1^ The original equations in a steady-state undepleted pump approximation read
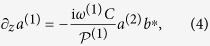

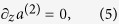

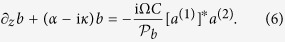


The distinction between forward and backward SBS is made by the sign of the Stokes wave’s modal power flux 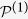; positive and negative values correspond to forward and backward propagation, respectively. The acoustic equation involves a parameter *κ* = *β*^(1)^ − *β*^(2)^ + *q*, which is the mismatch of the optical wave numbers *β*^(1,2)^ and the acoustic wave number *q* and is needed to study the shape of the SBS resonance^18^. In the following, we assume that the waveguide starts at *z* = 0 and ends at *z* = *Z*. We also make the approximation that the acoustic amplitude drops to zero at the beginning and the end of each waveguide section. In reality the acoustic amplitude will drop more smoothly, depending on the junction at the end of the waveguide segment and on the materials involved. Here we consider the situation where the transition is sufficiently abrupt that the majority of the acoustic mode is lost within a small fraction of the scale of variation of the acoustic envelope within the waveguide.

First, we recall the solution for a local acoustic response. This is the common expression for long waveguides which holds when the typical gain length far exceeds the decay length 1/*α*, and is based on the approximation
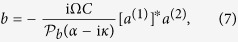
which implies
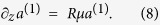
Here, we have introduced for the sake of brevity
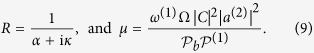
Consequently, *μ* > 0 describes forward SBS configurations, whereas *μ* < 0 describes backward SBS. For both cases, the solution to Eq. (8) is simply
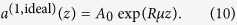
The constant *A*_0_ is the Stokes amplitude at position *z* = 0, proportional to the square root of input power in the case of forward SBS and the output power in the case of backward SBS. The real part of the product *Rμ* in Eq. (8) describes how quickly the Stokes amplitude grows whereas its imaginary part describes the evolution of the optical phase. The real part is proportional to 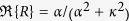, which means that the SBS resonance takes the form of a Lorentzian with respect to the detuning parameter 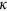, as one would expect.

Next, we solve the system formed by Eqs. (4, 5, 6) without further approximations such as Eq. (7). To this end, we take the 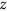-derivative of Eq. (6) and insert Eq. (4) to obtain
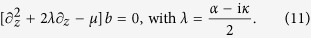
The two solutions to this equation (the degenerate case Λ = 0 corresponds to unrealistically high SBS gains and is ignored) have the form
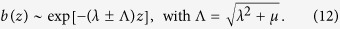
Since the waveguide section starts at *z* = 0, and the phonon propagation constant *q* is positive, we assume the initial condition *b*(0) = 0. This leads to the solution for the acoustic part of the problem:
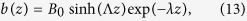
where *B*_0_ is a constant that is ultimately determined by the power of the incoming Stokes mode. The regimes Λ/*λ* > 1 and Λ/*λ* < 1 correspond to forward and backward SBS respectively. Note that even in high-gain SBS devices, we can expect |*μ*| < *λ*^2^.

## Discussion

**Figure 2:**
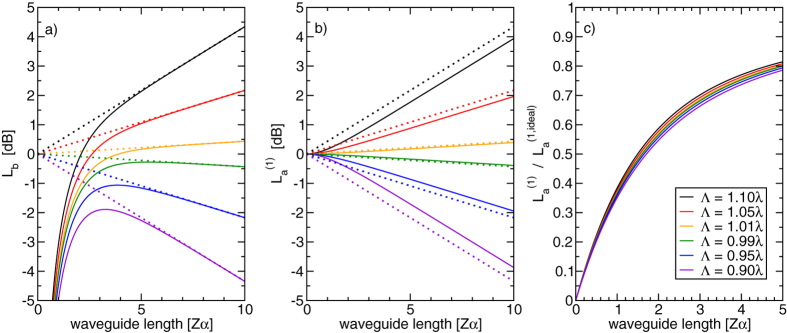
Behavior of the acoustic power level *L*_*b*_ = 10 log_10_ (|2*b*|^2^/|*B*_0_|^2^) normalized to the naively expected power level at *z* = 0 (panel a), the Stokes power level 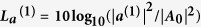 (panel b) and the gain degradation (panel c) of a finite waveguide on resonance (*λ* = *α*/2) as a function of waveguide length Z in units of acoustic decay lengths *α*^−1^ in comparison with the naive expectation based on Eq. (10). Each set of lines with the same color represents the finite (solid lines) and idealized (dotted lines) behavior for one value of the parameter Λ (forward and backward SBS for Λ > *λ* and Λ < *λ*, respectively). The three panels show how the acoustic field approaches its nominal value only after several decay lengths whereas the SBS gain is systematically reduced to the value of an ideal waveguide that is shorter by roughly *α*^−1^. Note the dramatic and nearly linear gain reduction for *Zα* < 2, which is furthermore fairly independent of Λ.

Figure 2a shows the absolute value of Eq. (13) on resonance (*κ* = 0) for various values of *μ* (solid lines) together with corresponding values derived from Eq. (10) (dotted lines). Observe that at large 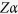, the phonon intensity evolves in accord with Eq. (10), growing exponentially in the positive direction for forward SBS (Λ > *λ*), and in the negative direction for backward SBS (Λ < *λ*). Note that the exponential growth exponent is slightly reduced for devices with extremely high gain, i.e. in the range *λ*^2^/10 < |*μ*| < *λ*^2^. This is a minor effect and will usually be negligible in comparison to the findings that we present in the following. For 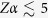, the phonon intensity is much reduced, indicating that several decay lengths are required for the phonon field to accumulate.

The optical Stokes amplitude *a*^(1)^ could be calculated by integrating Eq. (4) with an appropriate boundary condition. However, it is easier to obtain it directly from Eq. (6):
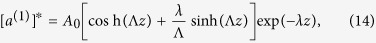
where *A*_0_ is again the Stokes amplitude at *z* = 0. Figure 2b shows the behavior of the absolute value of Eq. (14) on resonance both for forward and backward SBS (solid lines) again together with the corresponding curves that would be naively expected for a long waveguide according to Eq. (10) (dotted lines). Clearly, the Stokes amplitude is systematically reduced compared to the long waveguide approximation. (For the backward SBS cases, represented by the purple, blue and green lines, the predicted Stokes power shows as being larger than the ideal case (dotted lines). This is a consequence of our choice to display both forward and backward SBS curves with a common power at *Z* = 0: for BSBS, additional input Stokes power at the end of the waveguide, i.e. at *z* = *Z*, is required to achieve the common power at the waveguide’s front facet at *z* = 0.) The ratio between the logarithmic gains ( i.e. gains expressed in decibel) of the idealised and the finite waveguide (solid and dotted line with matching colors) is plotted in Fig. 2c. This is the degradation of the SBS gain due to the acoustic build-up. Its impact can be seen in an example: suppose that a waveguide with a length of *Zα* = 100 (so long that the acoustic build-up can be ignored) is divided into 50 shorter ones such that the acoustic amplitude is zero at the beginning of each one (e.g. by stabilizing a suspended waveguide with poorly designed posts every two decay lengths). Then, the gain of each short waveguide is about 0.6 (see Fig. 2c) of the gain of the corresponding section in the long waveguide. Since logarithmic gains simply add up, the total gain of the device is reduced by this factor due to the segmentation. To consider a specific example, a suspended silicon nanowire waveguide with an acoustic decay length of 50 *μ*m and an SBS gain of 5000 m^−1^ W^−1^, thereby having an expected gain of 2.8 dB over a length of 5 mm at a pump power of 26 mW, would have its gain reduced to 1.7 dB if the waveguide were split into 50 segments of 100 *μ*m each (corresponding to *Zα* = 2 in Fig. 2c). If the SBS-gain was now increased by reducing the acoustic loss inside the waveguide (say by increasing the decay length to 130 *μ*m tantamount to an SBS-gain of 1.3 × 10^4^m^−1^ W^−1^), the improved unsegmented waveguide would now provide the gain of 7.3 dB at 26 mW. However, the each segment in the cascaded design would now operate at *Zα* = 0.77, which leads to a gain penalty of about 0.28 resulting in only 2.0 dB, barely any different from the gain in the waveguide with lower acoustic quality factor.

**Figure 3:**
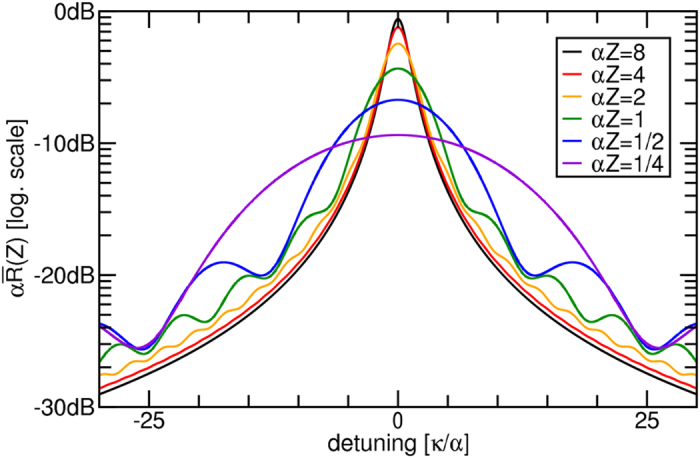
Resonance shape according to Eq. (16) for a wide range of waveguide lengths *Z* normalized to the peak gain of a long-waveguide approximation (0 dB-point). As the length is reduced to one acoustic decay length, the resonance broadens in a non-Lorentzian fashion, which becomes most prominent for *Z*α < 1. The graph values for various *Z* at the point 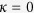 correspond to the values plotted in Fig. 2c for Λ ≈ 1.

Finally, one would expect that the SBS resonance broadens as the effective gain is clamped by the effect described above. We can see this from Eq. (14) by taking the logarithm of the Stokes amplitude at the waveguide’s end (*z* = *Z*) and dividing it by the factor *μZ*, i.e. the part of the exponent in Eq. (10) that does not depend on the Lorentzian *R*. This new quantity 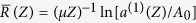 describes the SBS-gain distribution as a function of the detuning parameter *κ* and the waveguide length and is constructed to be directly compared to the ideal long waveguide resonance *R* in Eq. (8). We can see in Fig. 2c that the gain reduction is rather robust with respect to the coupling strength *μ*, so we may expect this to be true for the resonance shape as well. This justifies that we assume the term *μ*/*λ*^2^ to be small and approximate to leading order:
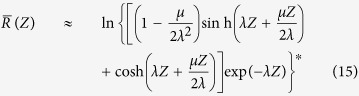

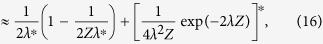
where we have used ln(1 + *x*) ≈ *x* in the second step. The leading term 1/(2λ^*^) = 1/(*α* − i*κ*) is exactly the Lorentzian resonance *R* that we expect for an infinitely long waveguide^18^. For a moderately long waveguide, the exponential remains negligible and the effective waveguide length is basically just reduced by one acoustic decay length *α*^−1^. For shorter waveguides, both the term 1/(2Zλ^*^) and the exponential term broaden the resonance and distort it into a non-Lorentzian shape as depicted in Fig. 3. As a consequence of the linear asymptotic behavior of the curves in Fig. 2c for small 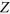, the gain of a short waveguide is not increased by reducing acoustic losses. Instead, the resonance line is deformed into a non-Lorentzian shape with a number of slowly decaying side bands and the total gain saturates at a maximum value. The result Eq. (16) was developed in a weak-gain approximation and is independent of the acousto-optic coupling. Small deviations can be expected in cases with strong interaction.

To illustrate the relevance of our findings in practical situations, consider backward SBS in a waveguide with an optical effective mode index of *n*_eff_ = 2 and a mechanical quality factor of *Q* = 1000 at a vacuum wavelength of *λ*_0_ = 1550 nm. The acoustic decay parameter *α* can be estimated in different ways. Firstly, it is related to the phonon life time *τ* via the acoustic group velocity *v*_*b*_: *α* = 1/(*v*_*b*_*τ*). Secondly, it can be estimated from the acoustic wave length and the mechanical Q-factor: The Q-factor describes the number of mechanical cycles in which the wave decays by 1/*e* (both with respect to cycles in time as well as cycles in space, i.e. the acoustic wave length). For backward SBS, the acoustic wave length is half the optical wave length in the waveguide, which in turn is related to the vacuum wave length via the effective mode index, so that
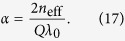
This yields an acoustic decay length of approximately α^−1^ ≈ 60*μ*m. The above-mentioned waveguide length of two acoustic decay lengths (resulting in a gain reduction to 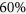) would correspond to 120 *μ*m, a very realistic length for silicon nanowires that are suspended in a wet etching process. The standard SBS gain derived from Eq. (8) suggests we can increase the gain by reducing the acoustic loss which is indeed intuitive. In this case, however, doubling the acoustic quality factor just increases the acoustic decay length to 120 *μ*m. According to Fig. 2c, the finite waveguide length will now reduce the SBS-gain to about 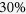 of the long-waveguide approximation, exactly cancelling any benefit from the reduced acoustic loss. This is an effect of the waveguide’s shortness, of course. It disappears if the waveguide is much longer than the acoustic decay length. This is also reflected by the fact that the curve in Fig. 2c deviates from the linear behavior for *Zα* > 2. For waveguides that are shorter than the acoustic decay length (60 *μ*m in this case), the non-Lorentzian line shape in Fig. 3 should begin to be observable provided that the SBS response can remain distinguished from the noise floor. As hinted at earlier, low acoustic loss in the suspended parts of the waveguide drastically increase the impact of the build-up effect and, therefore, lead to a stronger non-Lorentzian distortion.

In forward intra-mode SBS^13^ situations, the problems discussed in this paper are not relevant for two reasons: Firstly, the acoustic wavenumber is very close to zero, thence the acoustic wavelength is typically much longer than the waveguide. This means that the slowly varying envelope approximation for the acoustic mode is not justified. Consequently, the validity of the coupled mode equation (6) is debatable; the acoustic part behaves like a localized oscillator instead of a travelling wave. Secondly, the acoustic group velocity is extremely small, leading to a (hypothetical) acoustic decay length of less than a nanometer. However, our findings do apply to forward *inter*-mode SBS — i.e. scattering of co-propagating optical modes that live on different branches of the optical dispersion relation — provided that the branches of the dispersion relation are sufficiently far apart that the associated acoustic wavelength is smaller than the acoustic decay length. Such setups have recently attracted some attention for the development of all-optical isolators^19^,^20^.

In conclusion, we have demonstrated how the finite waveguide length can impose serious constraints to the design of integrated SBS-active devices both for backward-SBS and inter-mode forward-SBS. The results are of particular interest in the context of semiconductor nanowires but, clearly, also applicable to any other short type of waveguide.

## Additional Information

**How to cite this article**: Wolff, C. *et al.* Acoustic build-up in on-chip stimulated Brillouin scattering. *Sci. Rep.* **5**, 13656; doi: 10.1038/srep13656 (2015).

## Figures and Tables

**Figure 1:**
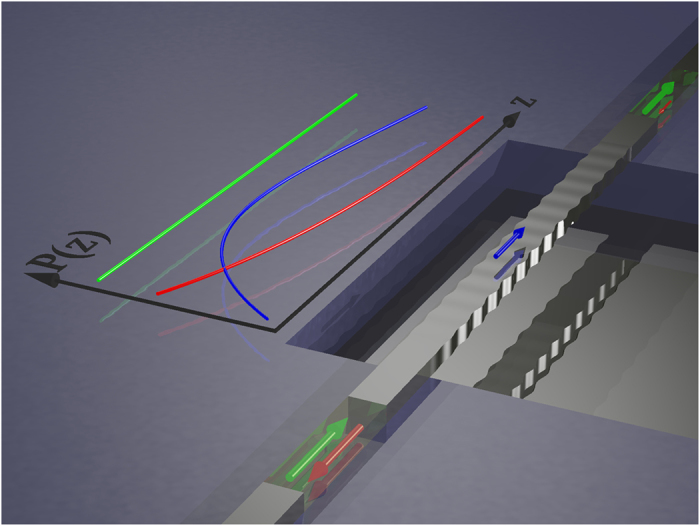
Qualitative illustration of the acoustic build-up effect in a suspended silicon waveguide. The arrows indicate power flux in the optical pump wave (green), the Stokes wave (red) and the acoustic wave (blue) in a short suspended backward SBS setup assuming weak pump depletion. The line graph to the left illustrates the evolution of the respective amplitudes (arbitrary units) throughout the waveguide. Note that the acoustic wave requires some length to build up, thereby reducing the overall SBS effect.
